# Efficacy and Safety of Sphincter-Preserving Surgery in the Treatment of Complex Anal Fistula: A Network Meta-Analysis

**DOI:** 10.3389/fsurg.2022.825166

**Published:** 2022-02-08

**Authors:** Hua Huang, Lijiang Ji, Yunfei Gu, Youran Li, Shanshan Xu

**Affiliations:** ^1^Department of Anorectal, Changshu Hospital Affiliated to Nanjing University of Chinese Medicine, Changshu, China; ^2^Department of Anorectal, Affiliated Hospital of Nanjing University of Chinese Medicine, Nanjing, China; ^3^Nanjing University of Chinese Medicine, Nanjing, China

**Keywords:** sphincter, treatment, complex anal fistula, meta, cure rate

## Abstract

**Background:**

There are many surgical methods of sphincter preservation in treating complex anal fistula, but the therapeutic effects of each operation are different. Therefore, this study aimed to compare the impact of other treatment methods through a network meta-analysis to evaluate the best sphincter preservation method for treating complex anal fistula.

**Methods:**

We searched PubMed, Embase, Cochrane Library, China National Knowledge Infrastructure, Chinese Biomedical Literature Database, VIP Journal Database, and the Wanfang Database to collate randomized controlled trials on sphincter-preserving surgery for complex anal fistula.

**Results:**

A total of 29 articles were included in this meta-analysis. The cure rates showed no statistically significant differences between any two interventions (*P* > 0.05). The recurrence rate results showed that the rate of patients after Fistulectomy was higher than others (*P* < 0.05). The incidence rate of complications showed that the incidence rate after fistulectomy treatment was higher than that of others (*P* < 0.05). The surface under the cumulative ranking (SUCRA) was used to arrange their advantages and disadvantages, and a larger SUCRA value indicates that the intervention may be more effective. The results showed that TROPIS may have the highest cure rate (SUCRA = 78.6%), stem cell transplantation (SCT) may have the lowest recurrence rate (SUCRA = 85.5%), and imLIFT may have the least complications (SUCRA = 88.2%).

**Conclusion:**

According to the existing literature data, for patients with complex anal fistula, TROPIS may be the surgical method with the highest cure rate, SCT may be the treatment method with the lowest recurrence rate, and imLIFT may be the surgical method with the lowest incidence of postoperative complications.

**Systematic Review Registration:**

PROSPERO, identifier: CRD42020221907.

## Introduction

A complex anal fistula is a refractory disease in colorectal anal surgery. According to statistics, the incidence of anal fistula is approximately 3.6%. Anal fistula mainly affects young adults with a male predominance (1, 2). An anal fistula is usually caused by an infection of the anal glands in the sphincter space. The passage of bacteria generally causes this infection into the anal recess. Clinical manifestations include anal pus and skin itching, amongst others. The condition seriously affects the quality of life of patients.

Surgery is the primary treatment method for complex anal fistula, with the main aim being to preserve anal sphincter function and eliminate the fistula. Traditional surgery requires an incision of healthy tissue and has certain shortcomings, including large drainage wounds, severe pain, slow healing, and varying degrees of damage to the anal sphincter (3, 4). Severe anal sphincter injury can lead to fecal incontinence (5).

It is crucial to preserve sphincter function in patients with complex anal fistula, and because traditional surgical methods easily injure the sphincter, a variety of surgical treatment modalities have been developed to preserve anal sphincter function, such as sphincter-preserving thread drawing (SPTD) (6), ligation of the intersphincteric fistula tract (LIFT) (7–9), valve displacement repair (VDR) (10, 11), fibrin glue (FG) (12), and biological patch (13, 14) treatments. Sphincter-preserving surgery for a complex anal fistula can maximize the maintenance of sphincter function and reduce postoperative complications. However, there are many types of sphincter-preserving treatment for high complex anal fistula, and the efficacy of each surgical treatment differs. At present, no treatment comparisons have been made. Therefore, this study evaluated various randomized controlled trials on treating complex anal fistula. A network meta-analysis compared differences in recurrence, cure rate, and complications of each sphincter-preserving therapy for patients with highly complex anal fistula to evaluate treatment safety and efficacy.

## Method

### Search Strategy

This network meta-analysis was registered in PROSPERO (Registration number: CRD42020221907), an international register website of systematic reviews (https://www.crd.york.ac.uk/prospero/) and was reported according to PRISMA (Preferred Reporting Items for Systematic Reviews and Meta-Analyses) (15) and AM- STAR (Assessing the methodological quality of systematic reviews) Guidelines (16).

We searched PubMed, Embase, Cochrane Library, China National Knowledge Infrastructure, Chinese Biomedical Literature Database, VIP Journal Database, and the Wanfang Database to collate randomized controlled trials on sphincter-preserving surgery for complex anal fistula from database establishment to July 31st, 2021. The languages were limited to Chinese and English. Search terms included “Stem Cell Transplantation”, “Sphincter preserving thread drawing”, “Biological patch”, “video-assisted anal fistula”, “Ligation of anal fistula”, “endoscopic needle-knife incision”, “anal fistula plug”, “Pushing mucosa”, “advancement flap”, “Transanal opening of intersphincteric space”, “Rectal Fistula”, “Anal Fistula” and “Complex”. Medical Subject Headings, free-text terms, and variants were used, including aliases for each surgery. Boolean Operators (AND, OR, and NOT) were used to connect the search terms to form search expressions.

### Eligibility and Exclusion Criteria

The inclusion criteria were as follows: (1) inclusion of patients with a definite diagnosis of complex anal fistula; (2) studies including interventions with various types of sphincter-preserving surgery; (3) study outcome indicators of cure rate, recurrence rate, and complication rate; (4) randomized controlled trials; (5) studies with complete data.

The exclusion criteria were as follows: (1) incomplete statistical analysis of results or insufficient data; (2) repeated published literature; (3) case report; (4) studies not examining sphincter-preserving treatment of complex anal fistula; (5) conferences, meta-analyses, and review articles.

### Quality Assessment and Data Extraction

Two investigators initially screened the retrieved studies independently according to the inclusion and exclusion criteria and then cross-checked. Controversial studies were evaluated by a third party and unified by discussion. Two investigators extracted relevant information from the included studies, including first author, publication year, publication country, sample size, age, sex, cure rate, recurrence rate, and complication rate.

Healing was defined as the absence of suppuration of the external orifice, and complete re-epithelialization was achieved after the end of follow-up. Recurrence occurred through the original tract and remained trans-sphincteric and was proven by clinical examination and ultrasound scanning. Complications refer to the occurrence of another disease or symptom during the treatment, and the latter is the complication of the former, which is not clearly defined in each literature.

Because the included studies were randomized controlled or cohort studies, the literature quality of randomized controlled trials was evaluated using the Jadad scale. The scores (0~3) were classified as low-quality literature and (4~7) as high-quality literature. The quality assessment of the included articles was performed using the Newcastle-Ottawa (NOS) scale, a quality evaluation tool specifically for case-control studies and cohort studies. The evaluation included three aspects: selection (four items), comparability (one item), and outcome (three items). Among them, the maximum score of each item of choice and outcome was 1, the total score of comparable items was 2, and the total score of scale evaluation results was 9. Scores (0–4) were classified as low-quality articles and (5~9) as high-quality.

### Statistical Analysis

Stata 16.0 software was used to analyze the data. Count data (binary data in this paper) are expressed by relative risk (RR), and the interval estimation used the 95% confidence interval (CI) as an indicator of affect quantity. Heterogeneity in the results was assessed using the Cochrane Q test (α = 0.1) combined with I^2^. If heterogeneity was acceptable, the fixed-effects model was used. Otherwise, the random-effects model was used. When the 95% CI did not contain “1,” the results were deemed statistically significant. If the 95% CI included “1,” this indicated no statistical significance. An overall inconsistency test was conducted when data were entered into Stata 16.0. If *P* > 0.05, overall consistency was good, and there was no statistically significant difference. Then, the consistent model was used to perform a network meta-analysis on the efficacy and safety of various sphincter-preserving surgeries. If there was a statistically significant difference (*P* < 0.05), non-consistency was used for model analysis. Using the node-splitting model, direct and indirect comparisons were compared. If *P* > 0.05, there was no apparent local inconsistency. Otherwise, there was local inconsistency between direct and indirect comparisons. After comparing various surgical methods, the advantages and disadvantages of each were arranged using the surface under the cumulative ranking (SUCRA), and the possibility of each type of anal sphincter preservation surgery as the best treatment was evaluated.

## Results

### Eligible Studies

**Figure 1:**
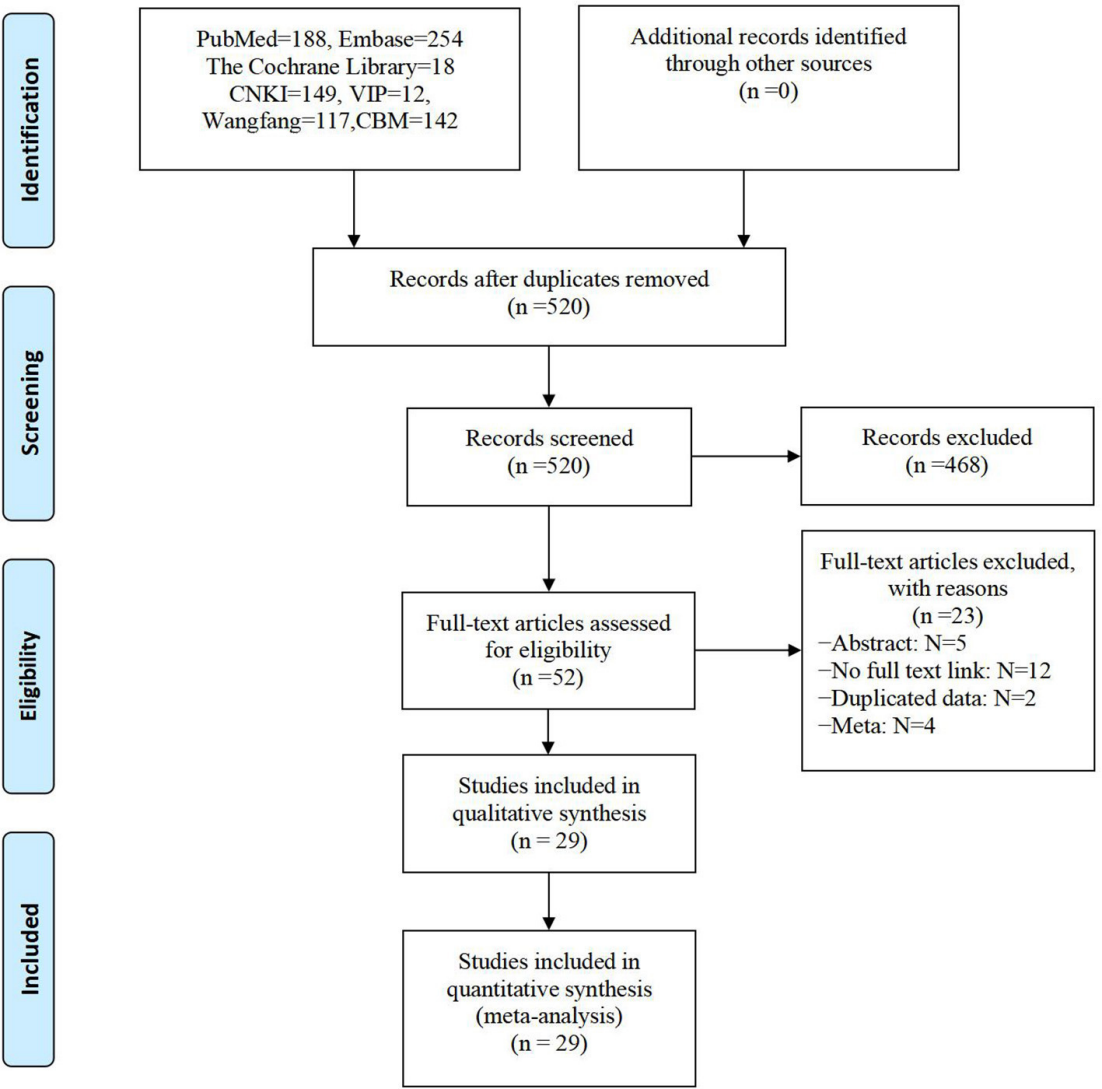
Literature screening flow chart.

A total of 880 relevant original articles were found in this reticular meta-analysis, including 460 English articles and 420 Chinese articles, involving 15 interventions. By carefully reading the titles and abstracts and screening the articles by inclusion and exclusion criteria, 52 articles were obtained and re-excluded by reading the complete text, and finally, 29 (17–45) articles were included in this study (Figure 1).

### Basic Characteristics and Quality Evaluation of Included Literature

The 29 included articles, with 3,608 patients, included 23 randomized controlled trial studies and six cohort studies. Only two pieces of literature in the randomized controlled trial study had low quality, and the rest had a Jadad score ≥ of 4 points. None of the cohort studies had a NOS score ≥5. Therefore, the overall quality of the included studies was good. The basic characteristics of the included studies are shown in Table 1.

**Table 1:** Basic characteristics and quality evaluation of the included studies.

**Study**	**Year**	**Country**	**Type of study**	**Age(s)**	**Gender (M/F)**	**Sample sizes**	**Interventions**	**Outcomes**	**Jadad/NOS scores**
Garcia-Arranz et al. (17)	2020	Spain	RCT	50.10 ± 10.7	16/7	23	SCTFG	➀➁	6
				50.86 ± 9.64	14/7	21	FG		
Garcia-Olmo et al. (18)	2009	Spain	RCT	42.64 ± 10.93	10/14	24	SCTFG	➀➂	5
				43.99 ± 8.97	14/11	25	FG		
García-Olmo et al. (19)	2015	Spain	RCT	42.64 ± 10.93	10/14	24	SCT	➁	7
				43.99 ± 8.97	14/11	25	FG		
Panés et al. (20)	2016	Spain	RCT	39·0 ± 13.1	60/47	107	SCT	➀➂	6
				37·6 ± 13.1	56/49	105	SOC		
Tsang et al. (21)	2020	China	RCT	47.2 ± 11.1	38/10	48	LIFT	➀➂	6
				47.2 ± 11.1	9/1	10	BioLIFT		
Liu H et al. (22)	2020	China	RCT	NA	54/10	64	LIFT	➀➁➂	6
				NA	52/12	64	SPTD		
Kun Gao et al. (23)	2018	China	RCT	44.19 ± 5.13	32/9	41	AF	➀➁➂	4
				43.21 ± 5.08	44/13	57	LIFT		
Junyi Jia et al. (24)	2017	China	RCT	46.51 ± 6.39	24/20	44	LIFT	➀➂	5
				46.82 ± 6.70	21/23	44	SPTD		
Tong Jia et al. (25)	2019	China	RCT	36.59 ± 9.28	32/9	41	AFS	➀➁	5
				37.98 ± 11.38	35/14	49	SPTD		
Linyuan Lu et al. (26)	2019	China	RCT	42.33 ± 2.76	34/8	42	VAAFT	➀➂	5
				42.29 ± 2.69	30/8	38	SPTD		
Jian Peng et al. (27)	2014	China	RCT	35.4 ± 8.7	25/15	40	LIFT	➀➁➂	6
				34.2 ± 8.5	23/17	40	SPTD		
Jinglin Wang et al. (28)	2018	China	RCT	38.94 ± 15.71	23/17	40	VAAFT	➀➂	3
				40.12 ± 16.33	21/19	40	SPTD		
Hongming Xu et al. (29)	2020	China	RCT	38.41 ± 9.58	35/12	47	imLIFT	➀➂	4
				38.07 ± 9.53	32/15	47	LIFT		
Changmou Yang et al. (30)	2007	China	RCT	38.7 ± 12.7	28/14	42	SPTD	➀➁➂	6
				41.9 ± 14.5	25/17	42	Fistulectomy		
Ming Ye et al. (31)	2014	China	RCT	NA	NA	37	SPTD	➀➁➂	3
				NA	NA	37	Fistulectomy		
Hexue Yuan et al. (32)	2019	China	RCT	44.3 ± 6.6	31/19	50	LIFT	➀➁➂	6
				46.4 ± 7.2	28/22	50	AF		
Le Zhao et al. (33)	2017	China	RCT	39 (22–52)	33/10	43	SPTD	➀➁	4
				42 (24–60)	35/12	47	IDBSS		
Li Zheng et al. (34)	2018	China	RCT	37.4 ± 13.5	33/9	42	VAAFT	➁	4
				42.1 ± 15.6	32/13	45	SPTD		
Junfeng Zhuang et al. (35)	2020	China	RCT	40.7 ± 5.2	25/32	57	ISDPS	➀	5
				40.2 ± 5.3	26/31	57	LIFT		
Yee Chen Lau et al. (36)	2019	Australia	RCT	38 (19–75)	68/37	105	LIFT	➀	6
				41 (26–69)	7/4	11	BioLIFT		
Chrispen Mushaya et al. (37)	2012	Australia	RCT	48.2 (20.6–72.9)	10/4	14	AF	➀➁➂	6
				47.5 (25.0–70.1)	17/8	25	LIFT		
M. D. Herreros et al. (38)	2012	Spain	RCT	49.78 ± 11.39	47/17	64	SCT	➀➁	6
				47.27 ± 12.27	36/24	60	SCTFG		
				50.85 ± 12.51	44/15	59	FG		
Wiley Chung et al. (39)	2009	Canada	Cohort study	46 (23~68)	18/9	27	FP	➀➁➂	5
				49 (22–68)	22/1	23	FG		
				46 (21–82)	70/16	86	SD		
				46 (28–75)	71/25	96	FA		
Oliver Maximilian Fisher et al. (40)	2015	Switzerland	Cohort study	41 (34–51)	17/14	31	AFS	➁➂	6
				44 (34–58)	29/11	40	AF		
A. Mujukian et al. (41)	2020	USA	Cohort study	35 (12–63)	16/22	38	LIFT	➀➁➂	6
				43 (22–68)	10/12	22	AFS		
M. La Torre et al. (42)	2020	Italy	Cohort study	NA	NA	26	LIFT	➀➁	5
				NA	NA	28	VAFFT		
Ian Lindsey et al. (43)	2002	Australia	RCT	NA	NA	13	FG	➀➁➂	4
				NA	NA	16	LIFT		
Pankaj Garg et al. (44)	2017	India	Cohort study	37.5 ± 10.7	510/101	611	Fistulectomy	➀➂	7
				40.5 ± 11.1	372/36	408	TROPIS		
				49.0 ± 10.9	52/4	56	AFS		
Zhiyun Zhang et al. (45)	2020	China	Cohort study	41.88 ± 13.38	18/7	25	Fistulectomy	➀➁➂	5
				41.12 ± 16.61	17/8	25	TROPIS		

*RCT, Randomized controlled trial; M, Male; F, Female; NA, Not available; SCTFG, Stem cell transplantation combined with fibrin glue; FG, Fibrin glue; SCT, Stem cell transplantation; SOC, Standard of care; LIFT, Ligation of intersphincteric fistula; BioLIFT, Biological patch combined with ligation of intersphincteric fistula; SPTD, Sphincter preserving thread drawing; AFS, Anal fistula suppository; VAAFT, Video-assisted anal fistula; VDR, Valve displacement repair; imLIFT, Improved ligation of intersphincteric fistula; IDBSS, Incision and drainage between sphincter and sphincter; ISDPS, Internal sphincterotomy and drainage with preservation of sphincter*.

*➀ Cure rate; ➁ Recurrence rate; ➂ Complication rate*.

### Results of Network Meta-Analysis

#### Evidence Network

**Figure 2:**
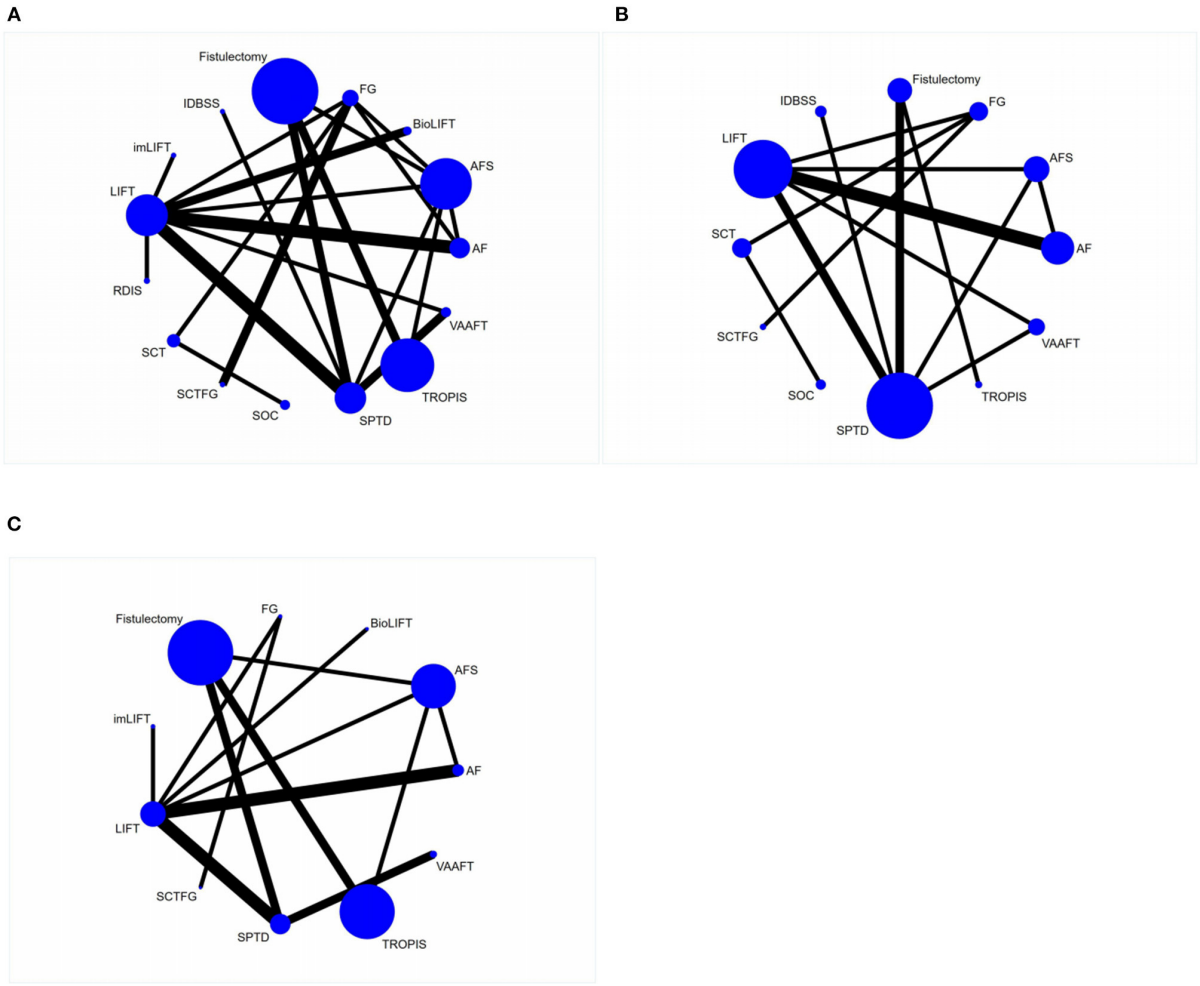
Reticulated evidence diagram of different sphincter-preserving surgeries. **(A)** Network evidence for cure rate; **(B)** Network evidence for recurrence rate; **(C)** Network evidence for complication rate.

In the reticulated evidence diagram, each vertex represents different intervention methods, the size of the vertex represents the sample size included in each intervention method, the line between vertices represents the direct comparison existing between two intervention methods, and the thickness of the line is directly proportional to the number of related studies. There was direct or indirect evidence between the different intervention methods, with the basic conditions for reticular meta-analysis (Figures 2A–C).

#### Network Meta-Analysis of Cure Rate

Twenty-six studies reported the cure rate of anal fistula. There was a closed ring between the interventions. There were direct and indirect comparisons between the interventions, and the results of the consistency test showed *P* > 0.05. Therefore, statistical analysis could be performed directly under the consistency model. The results of the network meta-analysis of cure rates showed that there were no statistically significant comparisons between any two interventions (*P* > 0.05) (Table 2).

**Table 2:** Network meta-analysis results of cure rate (RR, 95% CI).

AF														
1.58 (0.90, 2.78)	AFS													
1.24 (0.58, 2.68)	0.79 (0.34, 1.79)	BioLIFT												
1.24 (0.56, 2.72)	0.78 (0.34, 1.79)	0.99 (0.35, 2.81)	FG											
0.91 (0.46, 1.81)	0.58 (0.31, 1.08)	0.73 (0.30, 1.79)	0.74 (0.29, 1.87)	Fistulectomy										
1.04 (0.42, 2.59)	0.66 (0.27, 1.61)	0.84 (0.29, 2.42)	0.84 (0.27, 2.60)	1.14 (0.47, 2.77)	IDBSS									
0.79 (0.34, 1.86)	0.50 (0.20, 1.24)	0.64 (0.24, 1.72)	0.64 (0.21, 1.93)	0.87 (0.33, 2.28)	0.76 (0.25, 2.35)	imLIFT								
0.97 (0.64, 1.45)	0.61 (0.36, 1.02)	0.78 (0.41, 1.48)	0.78 (0.35, 1.75)	1.06 (0.58, 1.94)	0.93 (0.40, 2.16)	1.22 (0.58, 2.60)	LIFT							
0.77 (0.31, 1.90)	0.48 (0.19, 1.27)	0.62 (0.22, 1.74)	0.62 (0.20, 1.95)	0.84 (0.31, 2.32)	0.74 (0.23, 2.38)	0.97 (0.32, 2.94)	0.79 (0.35, 1.79)	RDIS						
1.13 (0.36, 3.56)	0.71 (0.22, 2.31)	0.91 (0.24, 3.44)	0.92 (0.40, 2.10)	1.24 (0.36, 4.32)	1.09 (0.27, 4.42)	1.43 (0.36, 5.69)	1.17 (0.37, 3.72)	1.48 (0.36, 6.05)	SCT					
0.79 (0.26, 2.35)	0.50 (0.16, 1.54)	0.63 (0.17, 2.31)	0.64 (0.30, 1.34)	0.86 (0.26, 2.84)	0.76 (0.19, 2.95)	0.99 (0.26, 3.80)	0.81 (0.27, 2.47)	1.02 (0.26, 4.05)	0.69 (0.23, 2.11)	SCTFG				
1.64 (0.40, 6.62)	1.03 (0.25, 4.28)	1.32 (0.28, 6.22)	1.32 (0.42, 4.20)	1.79 (0.41, 7.91)	1.57 (0.31, 7.91)	2.07 (0.42, 10.21)	1.69 (0.41, 6.91)	2.13 (0.42, 10.81)	1.44 (0.65, 3.22)	2.08 (0.53, 8.21)	SOC			
1.10 (0.64, 1.87)	0.69 (0.42, 1.14)	0.88 (0.41, 1.89)	0.89 (0.38, 2.08)	1.20 (0.74, 1.96)	1.06 (0.50, 2.22)	1.39 (0.59, 3.25)	1.14 (0.76, 1.69)	1.43 (0.58, 3.53)	0.97 (0.30, 3.18)	1.40 (0.44, 4.38)	0.67 (0.16, 2.81)	SPTD		
0.68 (0.28, 1.62)	0.43 (0.19, 0.97)	0.54 (0.19, 1.57)	0.55 (0.19, 1.57)	0.74 (0.41, 1.34)	0.65 (0.23, 1.87)	0.86 (0.28, 2.61)	0.70 (0.31, 1.59)	0.88 (0.28, 2.80)	0.60 (0.16, 2.29)	0.86 (0.24, 3.08)	0.41 (0.09, 1.98)	0.62 (0.29, 1.31)	TROPIS	
0.86 (0.45, 1.64)	0.54 (0.28, 1.04)	0.69 (0.30, 1.59)	0.70 (0.28, 1.77)	0.94 (0.48, 1.85)	0.83 (0.34, 2.00)	1.09 (0.44, 2.72)	0.89 (0.53, 1.50)	1.12 (0.43, 2.94)	0.76 (0.22, 2.65)	1.10 (0.33, 3.65)	0.53 (0.12, 2.32)	0.78 (0.49, 1.26)	1.27 (0.53, 3.06)	VAAFT

#### Network Meta-Analysis of Recurrence Rate

The recurrence rate was reported in 18 literature. There was a closed ring between the interventions. There were direct and indirect comparisons between the interventions. The consistency test results showed *P* > 0.05. Therefore, the statistical analysis could be performed directly under the consistency model. The results of network meta-analysis of the recurrence rate showed that the recurrence rate of patients after Fistulectomy treatment was higher than that of AF, AFS, LIFT, SCT, SCTFG, SPTD, and VAAFT, and the differences were statistically significant (*P* < 0.05); the recurrence rate of patients after FG treatment was higher than that of patients after SCTFG, and the differences were statistically significant (*P* < 0.05); the recurrence rate of patients after SOC treatment was higher than that of patients after SCT, and the differences were statistically significant (*P* < 0.05) (Table 3).

**Table 3:** Network meta-analysis results of recurrence rate (RR, 95% CI).

AF											
1.07 (0.63, 1.81)	AFS										
0.99 (0.17, 5.83)	0.93 (0.16, 5.51)	FG									
0.08 (0.01, 0.85)	0.08 (0.01, 0.80)	0.08 (0.01, 1.33)	Fistulectomy								
2.74 (0.10, 79.07)	2.57 (0.09, 74.11)	2.77 (0.07, 109.24)	32.58 (0.75, 1412.71)	IDBSS							
1.98 (0.97, 4.03)	1.86 (0.89, 3.86)	2.00 (0.39, 10.16)	23.55 (2.56, 216.52)	0.72 (0.03, 19.53)	LIFT						
8.89 (0.30, 263.52)	8.33 (0.28, 248.05)	8.98 (0.50, 161.47)	105.66 (1.96, 5698.76)	3.24 (0.03, 347.41)	4.49 (0.16, 123.32)	SCT					
7.59 (0.52, 110.77)	7.11 (0.48, 104.38)	7.67 (1.03, 57.22)	90.20 (2.99, 2719.25)	2.77 (0.04, 182.55)	3.83 (0.29, 50.77)	0.85 (0.03, 28.83)	SCTFG				
4.00 (0.13, 124.54)	3.75 (0.12, 117.22)	4.04 (0.21, 76.95)	47.54 (0.85, 2673.63)	1.46 (0.01, 162.00)	2.02 (0.07, 58.34)	0.45 (0.25, 0.80)	0.53 (0.01, 18.66)	SOC			
0.84 (0.28, 2.53)	0.79 (0.26, 2.37)	0.85 (0.13, 5.39)	9.96 (1.30, 76.02)	0.31 (0.01, 7.31)	0.42 (0.17, 1.03)	0.09 (0.00, 2.91)	0.11 (0.01, 1.70)	0.21 (0.01, 6.79)	SPTD		
0.34 (0.01, 7.77)	0.32 (0.01, 7.28)	0.34 (0.01, 10.95)	4.00 (0.48, 33.33)	0.12 (0.00, 9.28)	0.17 (0.01, 3.65)	0.04 (0.00, 3.46)	0.04 (0.00, 2.45)	0.08 (0.00, 7.99)	0.40 (0.02, 7.58)	TROPIS	
2.07 (0.82, 5.21)	1.94 (0.76, 4.95)	2.09 (0.37, 11.81)	24.58 (2.65, 228.34)	0.75 (0.03, 20.52)	1.04 (0.57, 1.91)	0.23 (0.01, 6.75)	0.27 (0.02, 3.87)	0.52 (0.02, 15.76)	2.47 (0.99, 6.16)	6.14 (0.28, 133.17)	VAAFT

#### Network Meta-Analysis of Complication Rate

The incidence rate of complications was reported in 18 pieces of literature. There were closed rings between the interventions. There were direct and indirect comparisons between the interventions. The consistency test results showed *P* > 0.05. Therefore, the statistical analysis could be performed directly under the consistency model. The results of network meta-analysis of the incidence of complications showed that the incidence of complications in patients after fistulectomy treatment was higher than that of AF, AFS, imLIFT, LIFT, SPTD, and VAAFT, and the differences were statistically significant (*P* < 0.05); the incidence of complications in patients after SPTD treatment was higher than that of imLIFT and LIFT, and the differences were statistically significant (*P* < 0.05); the incidence of complications in patients after TROPIS treatment was higher than that of imLIFT, LIFT, and AFS and the differences were statistically significant (*P* < 0.05); the incidence of complications in patients after VAAFT treatment was lower than that of SPTD and TROPIS (Table 4).

**Table 4:** Network meta-analysis results of patient complication rate (RR, 95% CI).

AF										
1.71 (0.61, 4.75)	AFS									
1.10 (0.14, 8.86)	0.64 (0.08, 4.96)	BioLIFT								
0.45 (0.01, 14.22)	0.26 (0.01, 8.11)	0.41 (0.01, 16.87)	FG							
0.14 (0.03, 0.75)	0.08 (0.02, 0.39)	0.13 (0.01, 1.19)	0.32 (0.01, 10.95)	Fistulectomy						
3.70 (0.71, 19.36)	2.17 (0.44, 10.71)	3.38 (0.39, 28.95)	8.27 (0.25, 272.27)	25.62 (4.29, 153.00)	imLIFT					
1.75 (0.57, 5.35)	1.03 (0.37, 2.87)	1.60 (0.27, 9.37)	3.92 (0.15, 103.46)	12.14 (3.29, 44.73)	0.47 (0.14, 1.61)	LIFT				
0.72 (0.02, 27.67)	0.42 (0.01, 15.81)	0.65 (0.01, 32.40)	1.60 (0.49, 5.21)	4.96 (0.12, 203.91)	0.19 (0.00, 7.74)	0.41 (0.01, 13.26)	SCTFG			
0.61 (0.15, 2.40)	0.36 (0.10, 1.29)	0.56 (0.08, 4.04)	1.36 (0.05, 40.59)	4.21 (1.50, 11.86)	0.16 (0.04, 0.75)	0.35 (0.14, 0.86)	0.85 (0.02, 30.96)	SPTD		
0.21 (0.03, 1.49)	0.12 (0.02, 0.78)	0.19 (0.02, 2.13)	0.47 (0.01, 18.28)	1.47 (0.51, 4.23)	0.06 (0.01, 0.44)	0.12 (0.02, 0.61)	0.30 (0.01, 13.76)	0.35 (0.08, 1.50)	TROPIS	
2.52 (0.43, 14.79)	1.48 (0.27, 8.10)	2.30 (0.24, 22.47)	5.64 (0.16, 201.31)	17.47 (3.82, 80.02)	0.68 (0.10, 4.50)	1.44 (0.34, 6.06)	3.52 (0.08, 152.09)	4.15 (1.36, 12.65)	11.88 (1.89, 74.52)	VAAFT

#### Probability Ranking of Intervention Effects of Various Surgical Methods

A total of 15 interventions were included in this study. The probability of cure rate, recurrence rate, complication rate and other indicators under 15 interventions was ranked. The probability indicated that the intervention was the best treatment. The results of probability ranking of cure rate showed: TROPIS (78.6%) > RDIS (68.3%) > imLIFT (66.9%) > SCTFG (66.3%) > VAAFT (64.8%) > Fistulectomy (58.4%) > LIFT (54.7%) > AF (51.1%) > IDBSS (47.8%) > SCT (44%) > SPTD (40.7%) > BioLIFT (34.5%) > FG (34.1%) > SOC (24.7%) > AFS (15.1%), suggesting that TROPIS may be the surgical method with the highest recovery rate in patients after treatment. The results of probability ranking of recurrence rate showed: SCT (85.5%) > SCTFG (83.7%) > SOC (66.2%) > VAAFT (65.5%) > LIFT (64.5%) > IDBSS (61.9%) > AFS (39.9%) > FG (38.1%) > AF (36.5%) > SPTD (31.7%) > TROPIS (24.2%) > Fistulectomy (2.4%), suggesting that SCT may be the surgical method with the lowest recurrence rate in patients after treatment. The results of probability ranking of complication rate showed: imLIFT (88.2%) > VAAFT (78.6%) > LIFT (69.1%) > AFS (68.8%) > BioLIFT (54%) > AF (49.7%) > SCTFG (47.9%) > SPTD (35.6%) > FG (34.2%) > TROPIS (16.3%) > Fistulectomy (7.6%), suggesting that imLIFT may be the surgical method with the lowest complication rate in patients after treatment (Table 5).

**Table 5:** Ranking of probabilities for each intervention (SUCRA, %).

**Interventions**	**Cure rate**	**Recurrence rate**	**Complication rate**
AF	51.1	36.5	49.7
AFS	15.1	39.9	68.8
BioLIFT	34.5	-	54.0
FG	34.1	38.1	34.2
Fistulectomy	58.4	2.4	7.6
IDBSS	47.8	61.9	-
imLIFT	66.9	-	88.2
LIFT	54.7	64.5	69.1
RDIS	68.3	-	-
SCT	44.0	85.5	-
SCTFG	66.3	83.7	47.9
SOC	24.7	66.2	-
SPTD	40.7	31.7	35.6
TROPIS	78.6	24.2	16.3
VAAFT	64.8	65.5	78.6

#### Node Analysis

The inconsistency test of cure rate, recurrence rate, and incidence rate of complications showed *P* > 0.05, indicating no significant inconsistency. The node analysis results showed no significant difference between direct comparison and indirect comparison (*P* > 0.05), indicating no inconsistency in the results between direct comparison and indirect comparison.

#### Small Sample Effect and Publication Bias

**Figure 3:**
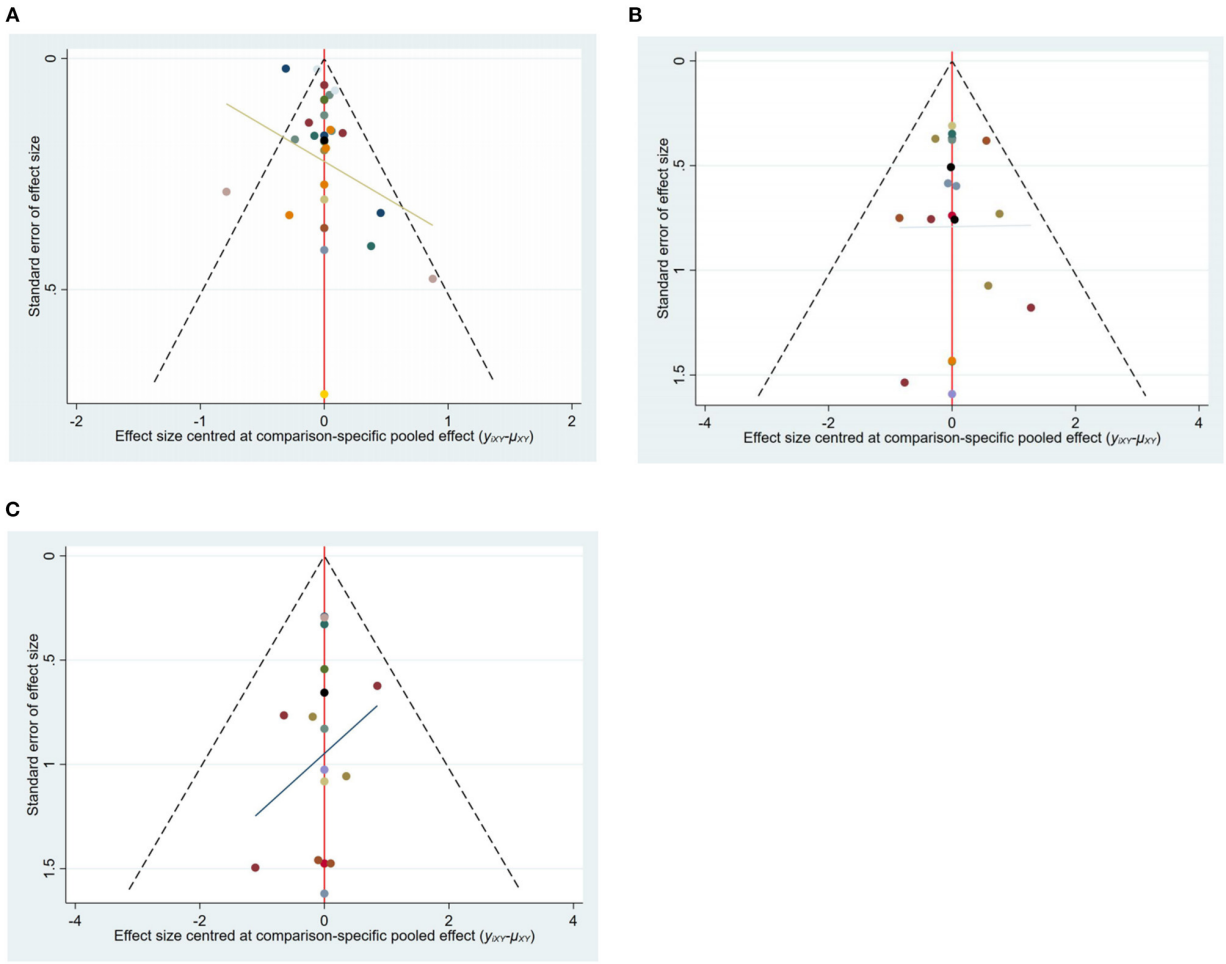
The funnel plot of outcome measures. **(A)** Funnel plot for cure rate; **(B)** Funnel plot of recurrence rate; **(C)** Funnel plot of complication rate.

The funnel plot of outcome measures such as cure rate, recurrence rate, and complication rate was plotted. From the funnel plot of cure rate, recurrence rate, and complication rate, most studies' scatter points were located above the funnel plot. The distribution of each issue was symmetrical, indicating that the included studies had less possibility of publication bias. At the bottom of each funnel plot, some scatter points are located at the bottom of the funnel plot, indicating that it is affected by some small sample effect (Figures 3A–C).

## Discussion

An anal fistula is a chronic abnormal sinus tract formed after ulceration of perianorectal abscess. The fistula of complex anal fistula has a complicated course, high recurrence rate, and partial loss of anal function, which is still one of the difficult problems in surgical treatment. Preservation of the patient's anal sphincter function is directly related to the quality of life later. For this reason, a variety of surgical treatments with anal sphincter preservation have been used in clinical practice.

Different treatment modalities vary in postoperative cure rate, recurrence rate, and complication rate. The drainage thread-drawing method allows the fistula to be in a continuous opening with adequate drainage to avoid recurrent episodes of the fistula and accelerate the epithelialization of the wall. However, some studies (46) have reported that the recurrence rate of anal fistula treated with thread-drawing therapy can be up to 40%. Women are more likely to experience treatment failure due to anal canal stenosis, rectovaginal fistulas, and complex fistulas. Fibrin glue is composed of fibrin and thrombin. After the mixture of the two is injected into the fistula, thrombin is activated to form a fibrous clot, which mechanically closes the fistula. Subsequently, the fibrous clot gradually dissolves to promote tissue healing and eliminate the fistula.

According to studies (47, 48), fibrin glue is well tolerated by patients in the treatment of anal fistula without the risk of anal incontinence. Still, its effect in treating complex anal fistula is not satisfactory, with a cure rate of <10%. For patients who did not respond to fibrin glue for the first time, there was no response after retreatment with fibrin glue, indicating that fibrin glue is not suitable for local conditions in patients who failed fibrin glue for the first time (49). An anal fistula plug is a suppository made of biological collagen extracted from the submucosa of the lyophilized pig small intestine. Anal fistula plug provide a reticular scaffold structure for host tissue cell growth and promote local tissue repair. A prospective, multi-center, randomized controlled study included 106 patients with anal fistula caused by Crohn's disease. After treatment for 12 years, the efficacy of thread-drawing therapy was similar to that of anal fistula plug, without significant difference. However, it was found that the effectiveness of the anal fistula plug was due to thread-drawing in patients with complex Crohn's disease anal fistula (50). Recurrence after treatment of anal fistula plug may be due to displacement of anal fistula plug, incomplete closure of the internal orifice, or multiple fistulas. Although the effect of anal fistula plug treatment is general, it also has certain advantages, such as simple operation, minimally invasive, fewer complications, and not easy to cause anal incontinence.AF uses a mucosal flap to cover the high-pressure area of the internal orifice and form a firm anti-infective barrier to promote fistula healing. Theoretically, AF can protect the normal anatomy of the anal canal and anal continence function, but 9.4 to 23.5% of patients have incontinence symptoms, which may be due to intraoperative damage to the internal anal sphincter or postoperative mucosal eversion, abnormal stimulation of anal defecation receptors, resulting in incontinence symptoms (51, 52).VAAFT mainly includes anal fistula endoscopy, fistula ablation, and internal orifice closure technique so that the internal orifice is closed, which can cure the fistula without damaging the anal sphincter. There were some differences in the success rate of VAAFT in the treatment of patients with anal fistula. Minero et al. (53) found that the cure rate was up to 87.7%, but Seow-En et al. (54) concluded that the primary healing rate was 70.7%.LIFT is the mainstream treatment for transsphincteric anal fistula, which can effectively avoid sphincter injury and is often used to treat refractory or recurrent anal fistula. The incision of conventional LIFT is close to the medial side of the anal verge, small and deep, which quickly leads to effusion or hematocele, which induces postoperative incision dehiscence, infection, and increases the risk of recurrence. In recent years, to achieve a better therapeutic effect, some new treatment methods continue to emerge, and researchers continue to report the efficacy of new treatment options.

Due to the differences and wide variety of measures for treating high complex anal fistula, there is no comparative analysis of the efficacy of different anal sphincter-preserving treatment measures. Therefore, this study is the first indirect comparison of different anal sphincter-preserving outcomes using network meta-analysis. In this meta-analysis, TROPIS was the treatment with the highest cure rate. As a newly used regimen in recent years, TROPIS has been confirmed to have an excellent therapeutic effect in several studies (44, 45). The surgical steps of TROPIS are mainly explored by using a probe at the external orifice. Then, based on the probe direction, a radioactive shuttle incision about 2.5 cm in length and perpendicular to the internal orifice is made to completely expose the internal and external sphincters, as well as the central space, a slight texture is used in the sphincter space to separate the internal and external sphincters; the probe is gradually elicited from the inner orifice, a rubber band is used to determine the tightness by the cumulative number of sphincters, and then the fibrotic wall tissue is trimmed (3). The infected anal glands and mucosa on both sides of the internal orifice are treated. After the internal orifice and infected anal glands are cleaned, the curette is used to curette the necrotic tissue in the fistula tract of the patient. Under appropriate circumstances, the lower part of the external sphincter and the superficial part can be removed to ensure patient drainage patency. Although TROPIS showed a higher cure rate, it did not perform very well in reducing the recurrence rate and complication rate. In terms of reducing the recurrence rate, stem cells have potent and immunomodulatory effects, differentiate into fibroblasts, and promote wound healing, an emerging method for treating complex anal fistulas. A multi-center phase I/IIa clinical trial initially reported 24 weeks of allogeneic adipose-derived stem cell transplantation for anal fistula in Crohn's disease, with an external orifice closure rate of 56% (55). Stem cell transplantation for patients with anal fistula has no serious adverse effects, and anal pain is one of the most common manifestations (20). The modified LIFT also ranked highest in reducing the complication rate. The surgical incision of modified LIFT is adjusted from the intersphincteric sulcus of the medial anal linea alba of the anal verge to the external orifice of the fistula. The external orifice is centered on keeping the incision away from the anal orifice to reduce the infection caused by feces entering the incision, reduce the risk of hematocele, effusion, and wound dehiscence. Perform tunnel resection of the fistula from the external orifice, stealth dissects the fistula to the intersphincteric sulcus and suture, seal the fistula and the internal orifice, and thoroughly dissect the fistula and suture the part of the internal orifice of the fistula to avoid residual necrotic tissue in the wall. For patients with long fistulas, a segmented incision can be made for tunnel sneak resection of the fistula. Suture the intersphincteric groove musculature and surgical incision, indwell multi-side hole negative pressure drainage tube for timely drainage of excess wound exudation, and compression bandaging at the incision skin during dressing change can promote adhesion and improve the wound healing rate.

### Limitations of This Study

(1) There are few direct comparison studies among various interventions, and few closed rings are formed. The results mainly come from indirect comparison. Although the indirect comparison results have specific guidelines, the strength of evidence is weaker than direct comparison; (2) There are still few relevant studies reporting the postoperative pain level of patients with anal sphincter-preserving surgery for anal fistula. This Meta has not evaluated the tolerance of patients.

## Conclusion

In the present study, it was found that TROPIS may be the treatment with the highest cure rate, SCT may be the treatment with the lowest recurrence rate, and imLIFT may be the surgical modality with the minor postoperative complications. Since the conclusion of this study is mainly derived from the results of the indirect comparison, it is hoped that the subsequent randomized controlled trial with rigorous protocol can be designed for further demonstration to provide better strong evidence support and guidance for the clinical treatment of patients with recurrent anal fistula.

## Data Availability Statement

The original contributions presented in the study are included in the article/supplementary material, further inquiries can be directed to the corresponding author.

## Author Contributions

HH: study design, data collections, and writing. YL and SX: data collections, data analysis, and writing. LJ: funding and study design. YG: study design and review. All authors contributed to the article and approved the submitted version.

## Funding

This study was funded by Changshu Health and Family Planning Commission Supporting Project (Grant/Award Number: csws201703), Changshu Health Committee Project (Grant/Award Number: cswsq202007), National Natural Science Youth Fund Project (Grant/Award Number: 81904204), Jiangsu Youth Science Foundation Project (Grant/Award Number: bk20191091), and Project on Academic Talents of Affiliated Hospital of Nanjing University of Chinese Medicine (Grant/Award Number: y2021rc35).

## Conflict of Interest

The authors declare that the research was conducted in the absence of any commercial or financial relationships that could be construed as a potential conflict of interest.

## Publisher's Note

All claims expressed in this article are solely those of the authors and do not necessarily represent those of their affiliated organizations, or those of the publisher, the editors and the reviewers. Any product that may be evaluated in this article, or claim that may be made by its manufacturer, is not guaranteed or endorsed by the publisher.
